# Exposing Digital Image Forgeries by Detecting Contextual Abnormality Using Convolutional Neural Networks

**DOI:** 10.3390/s20082262

**Published:** 2020-04-16

**Authors:** Haneol Jang, Jong-Uk Hou

**Affiliations:** 1 Cyber Security Research Division, The Affiliated Institute of ETRI, Daejeon 34044, Korea; hejang@nsr.re.kr; 2 School of Software, Hallym University, Chuncheon 24252, Korea

**Keywords:** digital image forensics, contextual abnormality, deep learning, R-CNN, localization

## Abstract

Traditionally, digital image forensics mainly focused on the low-level features of an image, such as edges and texture, because these features include traces of the image’s modification history. However, previous methods that employed low-level features are highly vulnerable, even to frequently used image processing techniques such as JPEG and resizing, because these techniques add noise to the low-level features. In this paper, we propose a framework that uses deep neural networks to detect image manipulation based on contextual abnormality. The proposed method first detects the class and location of objects using a well-known object detector such as a region-based convolutional neural network (R-CNN) and evaluates the contextual scores according to the combination of objects, the spatial context of objects and the position of objects. Thus, the proposed forensics can detect image forgery based on contextual abnormality as long as the object can be identified even if noise is applied to the image, contrary to methods that employ low-level features, which are vulnerable to noise. Our experiments showed that our method is able to effectively detect contextual abnormality in an image.

## 1. Introduction

Digital images have become one of the most popular information sources in the age of high-performance digital cameras and the Internet. Unlike textual information, images offer an effective and natural communication medium for humans, because the visual nature of images facilitates an effective understanding of the content. Traditionally, the integrity of visual data was accepted with confidence, such that a photographic image in a newspaper was commonly accepted as a certification of the news. Unfortunately, digital images are easily manipulated, especially since the advent of high-quality image-editing tools, such as Adobe Photoshop and Paintshop Pro. Moreover, as a consequence of the invention of generative adversarial networks [1], deepfake technology has been posing a threat to the reality and integrity of image media [2] because this technology can easily generate photo-realistic fake images. As a result, digital-image forensics, a practice aimed at identifying forgeries in digital images, has emerged as an important field of research.

A number of forensic schemes were proposed recently to detect image forgeries. Most previous digital image forensic methods [3,4] focused on low-level features that include traces of the image modification history. Pixel photo response non-uniformity (PRNU) is also widely used for detecting digital image forgeries [5,6,7,8]. Methods for detecting identical regions caused by copy–move forgery [9,10,11] have also been developed. Approaches based on deep neural networks have also delivered remarkable results. These approaches mostly focus on detecting local inconsistencies or statistical features in images that have been tampered with [12,13,14,15,16].

**Figure 1:**
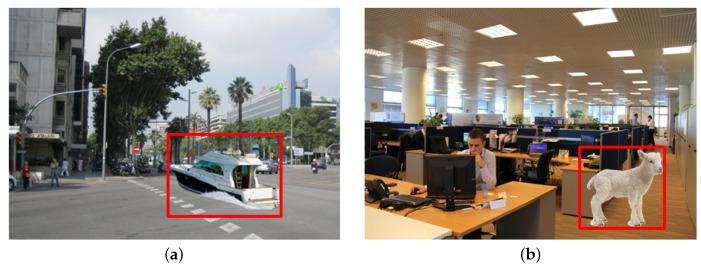
Examples of image forgery detection based on contextual abnormality. The manipulated parts (areas in the red box) can be detected because these objects caused contextual abnormalities. (**a**) a boat surrounded by cars and trees constitutes a contextual abnormality, (**b**) the presence of the lamb in the office is quite an unusual situation.

In this paper, we present a forensic scheme to detect whether images have been manipulated. The proposed scheme detects contextual abnormality based on high-level objects in the target image. For example, as shown in Figure 1a, the presence of the lamb in the office is quite an unusual situation. Figure 1b, which shows a boat surrounded by cars and trees constitutes a contextual abnormality. The manipulated parts (areas in the red box) can be detected because these objects caused contextual abnormalities.

The work presented in this paper follows the concept we previously reported [17], which describes a context-learning convolutional neural network (CL-CNN) that detects contextual abnormality in an image. Here, we add detailed explanations and data that were not included in our previous paper. The information about the relationship between objects in an image is used as a robust feature that is not affected by general image processing. The context that frequently occurs in the original image has a high contextual score. A context abnormality is a case that contains unexpected object combinations or unexpected spatial context, and the context abnormality can be measured by the contextual score. To measure the image context described above, various learning models for object relations have been proposed. Existing context models [18,19,20] operate with co-occurrence statistics based on a tree, and a graph-structured model. However, existing models mainly focus on co-occurrence; thus, the opportunity to learn the spatial context is limited.

We propose a model that can identify the context by learning the image label that contains the object class and the spatial coordinate. The main contributions of our approach are the context-learning convolutional neural networks (CL-CNN), and the digital forgery detector in combination with CL-CNN and the trained object detector, and more specific descriptions are as follows.

- We propose a CL-CNN that detects contextual abnormality in an image. The CL-CNN was trained using a large annotated image database (COCO 2014), and was then used to learn the spatial context that was not provided by the existing graph-based context models.
- In combination with a well-known object detector such as a region-based convolutional neural network (R-CNN), the proposed method can evaluate the contextual scores according to the combination of the objects in the image, and the spatial context among the objects. The proposed detector in combination with CL-CNN and the object detector can detect image forgeries based on contextual abnormality as long as the objects in the image can be identified by the R-CNN, in contrast to the family of low-level feature-based forgery detectors.

## 2. Related Work

### 2.1. Image Forensics

Image forensics algorithms can be categorized in many ways. In this section, we categorize detectors into those with low-level features and those with high-level features. Previously, digital image forensics [3,4] mostly focused on low-level features that include traces of the image modifying history. These methods are designed to detect local inconsistencies such as resampling artifacts [21], color filter array interpolation artifacts [22], camera response function [23,24] and JPEG compression [25]. Pixel photo response non-uniformity (PRNU) is another family of low-level feature-based detectors, which is widely used for detecting digital image forgeries [5,6,7,8] and camera source identification [5]. Approaches based on deep neural networks have also been used to investigate low-level image features. Methods that focus on low-level features mostly focus on detecting local inconsistencies or statistical features relating to the manipulated images [12,13,14,15,16].

**Figure 2:**
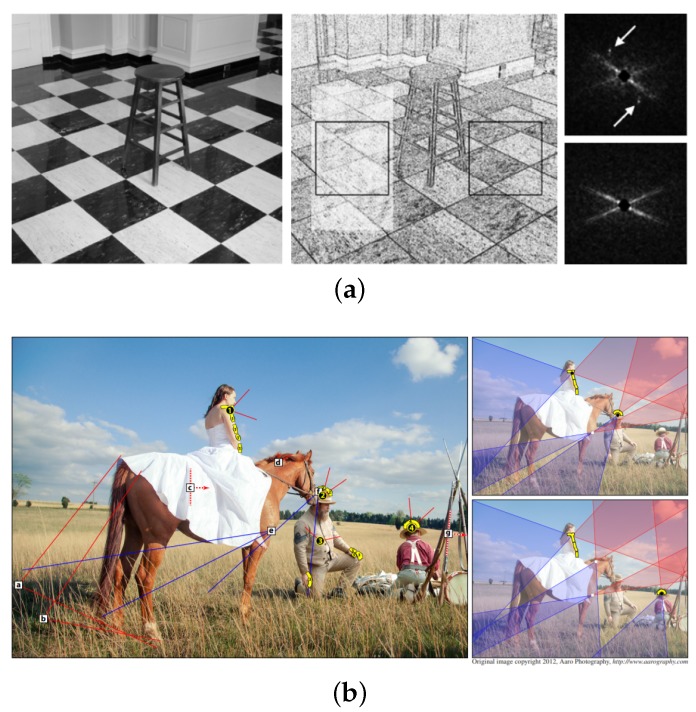
(**a**) The low-level image features do not rely on the image contents that make them more consistent [21]. (**b**) The high-level feature provides reasonably robust clues for the forgery detection system against general image processing such as JPEG and scaling. One example is an image forgery detection based on shading and shadows [29].

Most of the methods based on detecting local inconsistencies are vulnerable to general image processing techniques such as JPEG and GIF compression, white balancing and noise addition. On the other hand, high-level features such as the lighting condition [26,27], inconsistent reflections [28] and shading and shadows [29] provide fairly robust clues for a forgery detection system against general image processing as shown in Figure 2. Compared to algorithms based on local inconsistencies, high-level features are not always effective in every case. However, successful identification of the artifacts of image manipulation in real examples rarely occurs because of the general use of image post-processing techniques such as JPEG and scaling.

### 2.2. Object Detection Based on Deep Learning

Object detection is the task of locating an object and its label in an image. A variety of object detectors based on deep neural networks have been developed. Because our experiments were conducted using faster R-CNN, we only review three related methods here. Girchick et al. [30] proposed R-CNN: Regions with CNN (convolutional neural networks) features to detect an object in an image. Their method applied high-capacity CNNs to carry out bottom-up region proposals to localize and extract objects. They also found that when the labeled training set is sparse, supervised pretraining for an auxiliary task followed by domain-specific fine-tuning results in significant performance improvement.

Although they improved the object detection task significantly, the framework has to process all of the proposals, thereby greatly increasing the computational cost. Therefore, Girchick et al. subsequently improved the detection speed [31] by developing a method named fast R-CNN. Instead of local proposals, the improved method processes the entire input image to extract the features. Ren et al. [32] presented faster R-CNN, which includes a region proposal network (RPN) that shares full-image convolutional features with the detection network. The RPN simultaneously predicts the object bounds and objectness scores at each position. In this study, we used the faster R-CNN to extract object labels and locations from a suspicious image.

## 3. Context-Learning CNN

In this research, we propose context-learning convolutional neural networks (CL-CNN) to learn the co-occurrence and spatial relationship between object categories. The work presented here follows the concept we previously reported [17], which describes a context-learning convolutional neural network (CL-CNN) that detects contextual abnormality in an image. The proposed CL-CNN provides an explicit contextual model by using deep learning with large image databases. The CL-CNN is trained to return a high value for the natural (or learned) combination of the object categories, whereas it returns a low value for atypical combinations or spatial contexts of the image labels.

### 3.1. Input Data Structure

**Figure 3:**
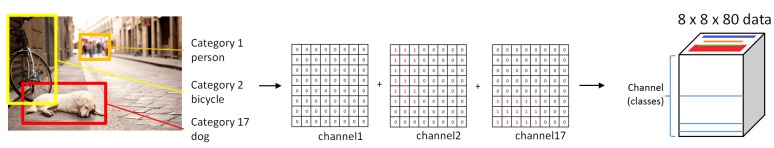
Input data structure and encoding process of the proposed method. Annotated images are used for providing labels and location information. The number of the channel of the input data structure is the total number of categories from the dataset.

The process of generating input data is as follows. We used an annotated image database for object location and category information. Because the size of the image is too large to be used, the size is reduced to N×N, where *N* is the height and width of the encoded data, to record the position information of the object. The channel size of the input data structure is the total number of categories that is necessary to distinguish each category. The part defining the label is padded with a value of 1 and the remaining blank area is filled with 0 values. This process is used to encode the location and label information into the input data structure as shown in Figure 3.

In this research, we reduced the size of the image to 8×8 and chose 80 as the category. The final input data size is 8×8×80 and the generation process is shown in Figure 3.

### 3.2. CL-CNN Structure

**Figure 4:**
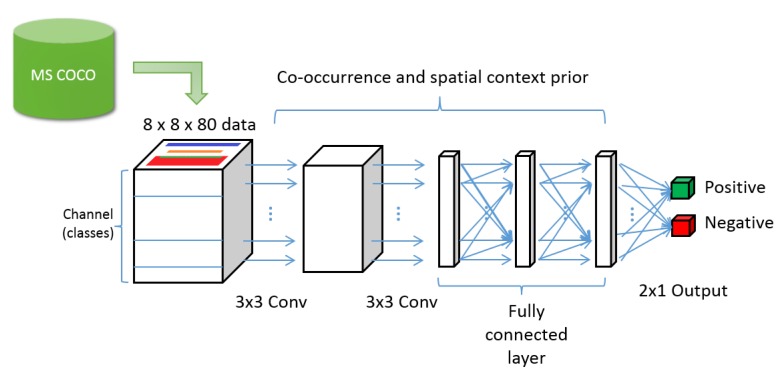
Overall structure of the context-learning convolutional neural networks. The network receives the encoded input data that consists location and label information. The input passes through two convolutional layers and three fully connected layers.

The structure of the CL-CNN is as follows (see Figure 4). It receives input data of 8×8×80, which pass through two convolutional and three fully connected layers. Then the fully connected layer finally outputs a 2×1 vector. The first value of the output is a score that evaluates the extent to which the label is natural in combination with the category and spatial context, and the second value is the score that evaluates the extent to which the category combination and spatial context of the label are awkward.

The loss function uses the Euclidean loss *L*, which is defined by

(1) $$L=\frac{1}{2}\sum_{i}{\left(y_{i}-a_{i}\right)}^{2},$$

where *y* is the output of the CL-CNN, and *a* is the label of the data sample.

### 3.3. Dataset Generation

A large number of datasets needs to be acquired to enable the proposed network to learn. We need both a collection of natural labels and a collection of unnatural labels. Moreover, we also need data that show both the location and type of the object. A dataset that meets these criteria is Microsoft COCO: Common Objects in Context [33]. Microsoft COCO 2014 provides 82,783 training image sets and label information and 40,504 validation images and label information. The dataset was therefore constructed to aid in learning detailed object models capable of precise 2D localization and contextual information.

Before we used the dataset, we excluded single-category images because they serve no useful purpose for learning contextual information. Thus, we used 65,268 multi-category images, which were divided into 80 categories, to train the CL-CNN. A positive set was constructed using label information of multi-category images, using the approach described in Section 3.1.

**Figure 5:**
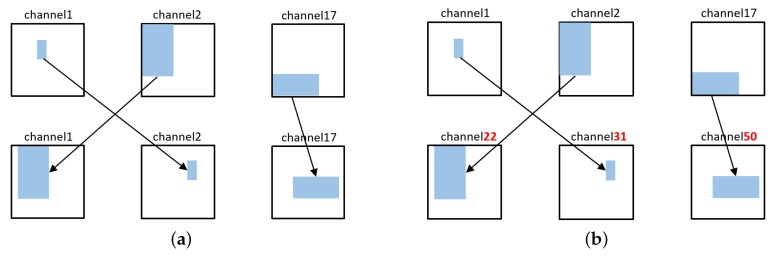
Sample illustration of the negative set generation. (**a**) Maintain category combination while changing each position; (**b**) change category combination with changing each position.

**Figure 6:**
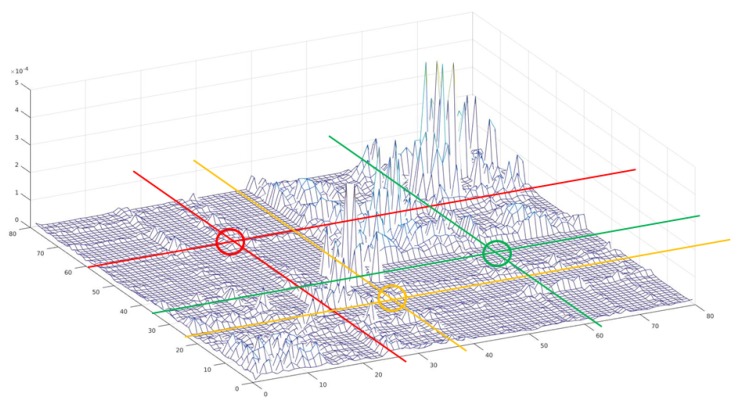
Histogram of the co-occurrences between object categories. The negative set was generated by selecting the combinations of the less correlated categories (e.g., combinations shown in circled regions).

We cannot use negative sets based on existing databases because they need to learn combinations of unnatural labels that do not actually exist. Therefore, we generated the negative set in two ways as shown in Figure 5. Negative set 1 was created by changing the size and position of the object while maintaining the category combination. Negative set 2 was created by selecting a combination of less correlated categories. Figure 6 shows the histogram of the co-occurrences between object categories. Using the probability $P(c_{1},c_{2})$ from the co-occurrence histogram, combinations of classes $c_{1},c_{2}$ with a low co-occurrence probability $P(c_{1},c_{2})$, were selected to generate a negative dataset. Next, negative set 2 was modified by changing the size and position of the object while maintaining the category combination.

### 3.4. Network Training

A simple learning approach was employed to train object combination and location shuffled datasets at the same time. Next, we tested “combination and location shuffling” and “location shuffling,” for which we obtained accuracies of 0.97 and 0.53, respectively. When learning “combination and location shuffling” at the same time, “combination change” was strongly learned. As a result, the spatial context was not learned because of overfitting of the CL-CNN. Therefore, we needed to improve the learning method to ensure that “location shuffling” was sufficiently learned.

Therefore, we trained the CL-CNN by learning the “location shuffling” of an object first and then fine-tuning the “combination and location shuffling” part sequentially. We set the learning rate to 0.001 for “location shuffling” and set the learning rate to 0.00001 for “combination and location shuffling” learning. We also tested “combination and location shuffling” and “location shuffling,” for which we obtained accuracies of 0.93 and 0.81, respectively. The test accuracy for “location shuffling” has been greatly improved from 0.53 to 0.81 when compared with the simultaneous learning approach.

## 4. Detection of Contextual Abnormality of Target Image

We propose a method to detect the contextual abnormality of the target image using CL-CNN. The proposed method functions in combination with the output of an existing object detector [30,31,32]. Ultimately, we selected the faster R-CNN [32] from among these methods to solve the object detection task. Using these object detection results and probability values, we developed a system that detects the objects that are most inappropriate in the image context. The proposed method is described as follows.

**Figure 7:**
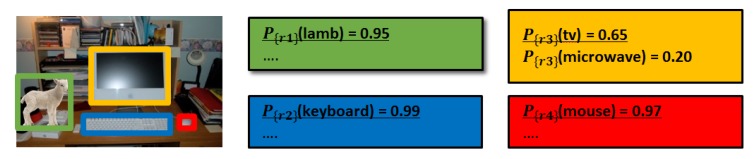
Step 1. Extract object region and calculate category score using the object detector such as the faster region-based convolutional neural network (R-CNN) [32].

**Step 1. Extract objects from the suspicious image:** Let *I* be a suspicious image. Using the image object detector, we extract the area containing the object in the image and calculate the category score in each area as follows:

(2) $$P_{r_{i}}\left(c\right)=F\left(I\right),$$

where the function F(·) is the region-based object detector such as faster R-CNN [32] for a single input image *I*. $P\in {\left[0,1\right]}^{R\times C}$ is the probability of each object class *c* from the detected region $r_{i}$. Figure 7 shows a sample of the object detection result and its details.

**Figure 8:**
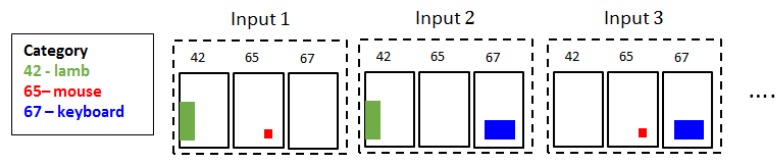
Generate input sets for context-learning convolutional neural networks (CL-CNN).

**Figure 9:**
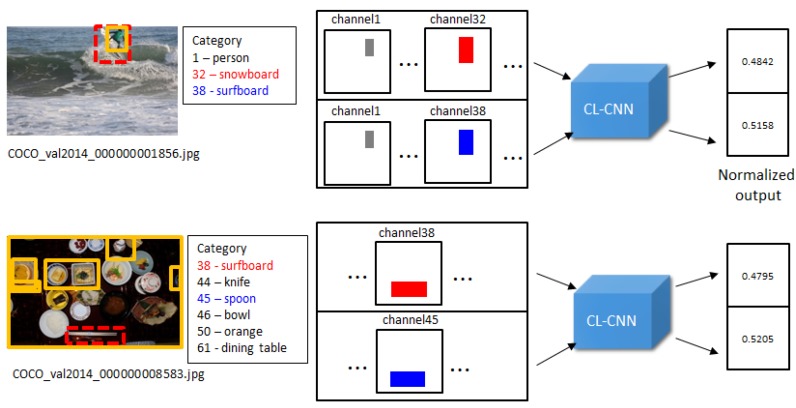
The encoded inputs from the detected objects by the R-CNN are processed through the CL-CNN. Each object from the output is respectively excluded as explained in Equations (4) and (5) to evaluate the contextual score.

**Step 2. Generate input sets for CL-CNN:** After extracting objects from the image, candidates for the contextual abnormality check are selected by:

(3) $$P=\{\left(r_{i},c\right):P_{r_{i}}\left(c\right)>\tau_{i}\},$$

where $\tau_{i}$ is the selection threshold for the raw output. If $P_{r_{i}}<\tau_{i}$, the corresponding object region $r_{i}$ is not used. For example, when $\tau_{i}=0.7$, three candidates: lamb, keyboard, and mouse are selected in the sample image in Figure 7. Then, the input sets $S_{i}$ for the CL-CNN are generated by:

(4) $$S_{i}=P\backslash \left\{\left(r_{i},c\right)\right\},$$

where P\{x} denotes the set P excluding the element *x*. As shown in Figure 8, each object is excluded from each of the generated input. For example, object 67 (keyboard) is excluded from the Input 1. Figure 9 shows that the encoded inputs from the detected objects by R-CNN are processed through CL-CNN. Each object from the output is respectively excluded as explained here to evaluate the contextual score.

**Step 3. Evaluate the context score of the inputs:** The encoded inputs from the objects detected by the R-CNN are processed by using the CL-CNN. Each respective object from the output is excluded as explained in Equation (5) to evaluate the contextual score. Each of the input sets $S_{i}$ is passed to the CL-CNN to generate the resultant value vector.

(5) $$\hat{i}=\underset{i}{\mathrm{argmin}}\left[C\left(S_{i}\right)\right],$$

where the return value of the function C(·) denotes the positive output value of the CL-CNN. Before calculating C(·), the input $S_{i}$ is converted according to the input data structure described in Section 3.1. Because $S_{i}$ is the set P that excludes the element $r_{i}$, the object class *c* from the region $r_{\hat{i}}$ is the most unlikely object in the context of the target image *I*. Therefore, $\hat{i}$ indicates the index value of the region that may cause the contextual abnormality.

In addition, we would need to consider the case in which the suspicious image does not contain contextual abnormality. Reduction of the false positive error requires us to verify whether the value of $C(S_{\hat{i}})$ is larger than the user defined threshold $\tau_{o}$.

(6) $$\left\{\begin{array}{cc} \mathrm{Forgery}\mathrm{detected}: & \;\;\;\;\;\;\;\;\;\;\;\;\mathrm{if}C\left(S_{\hat{i}}\right)<\tau_{o},\\ \mathrm{No}\mathrm{detection}: & \;\;\;\;\;\;\;\;\;\;\;\;\mathrm{otherwise}. \end{array}\right.$$

We use the value 0.2 for $\tau_{o}$.

The value of $C(S_{\hat{i}})$ indicates the confidence value of CL-CNN. If the positive output value $C(S_{\hat{i}})$ of CL-CNN is lower than $\tau_{o}$, the object from the detected region $r_{\hat{i}}$ is determined as a manipulated candidate by our detector. In cases in which multiple objects are forged, we can solve the above problem by checking the top *n* results of Equation (5). In other words, *n* most confident detections evaluated by CL-CNN were selected for the final result.

## 5. Experimental Results

### 5.1. Implementation Details

The experiment was conducted by using sample images collected from Microsoft COCO: Common Objects in Context [33]. We used 65,268 multi-category images, which were divided into 80 categories, to train the CL-CNN. A positive set was constructed using label information of multi-category images, using the approach described in Section 3.1. Two types of negative sets were synthesized as described in Section 3.3. The entire dataset was split to form the test and training set at a 9:1 ratio.

**Figure 10:**
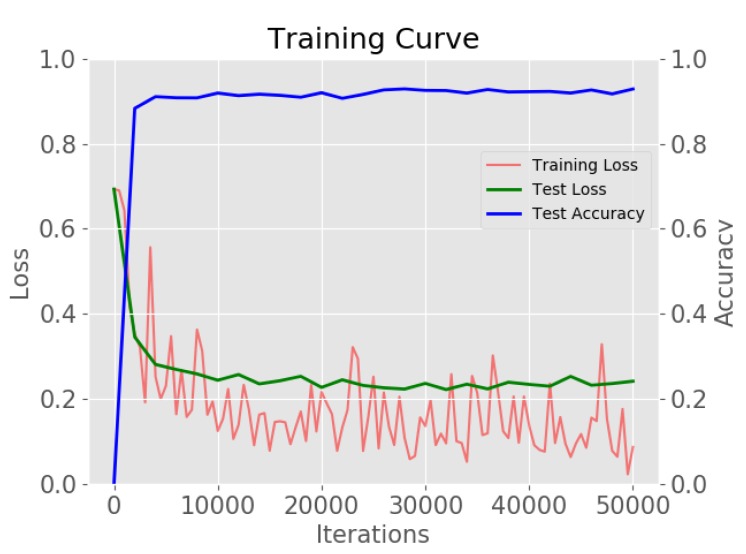
The overall training loss, test loss, and test accuracy of the proposed network.

The implementation of the CL-CNN is based on *Caffe library* [34]. The network was optimized using a stochastic gradient descent optimizer (SGD) and an inverse decay learning rate policy with the following hyper-parameters: gamma: 0.1, power: 0.75, weight decay: 0.0001, momentum: 0.9 and batch size: 64. The CL-CNN was trained a total of 50,000 iterations for the generated test set. We set the base learning rate to 0.001 for “location shuffling” and set the learning rate to 0.00001 for “combination and location shuffling” learning. Figure 10 demonstrates the overall training loss, test loss and test accuracy. The cross-entropy classification loss was used for the losses. To sum up, CL-CNN obtained a 92.8% overall accuracy for classifying the negative dataset from the original dataset.

### 5.2. Detection Results

**Figure 11:**
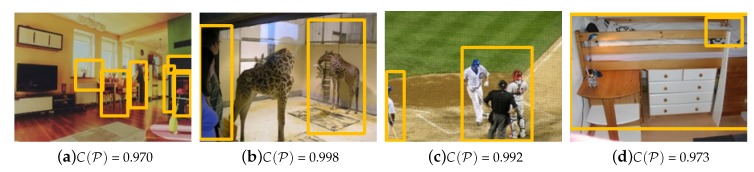
CL-CNN results with natural image input. The average output value was 0.98 or higher. The yellow box shows the normal detection results by R-CNN, and red box represent the detection result by our method. (**a**) indoor scene, (**b**) giraffes in the glass cage, (**c**) baseball scene, (**d**) indoor scene with shelf bed.

**Figure 12:**
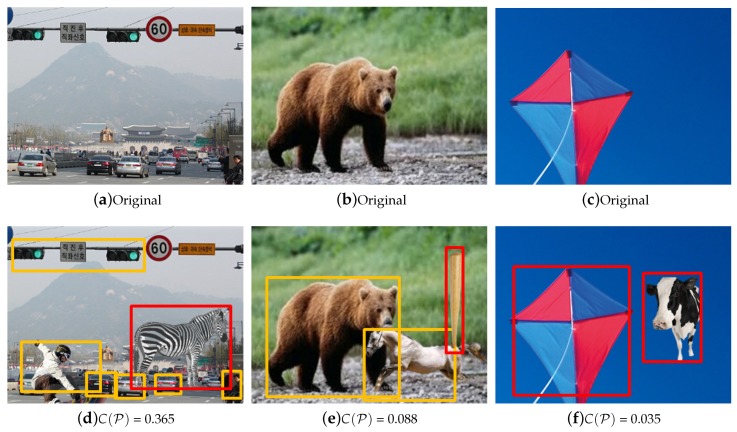
CL-CNN and forgery detection results with manipulated images. The object in the red box was detected as a manipulated object. The first row shows original images. The second row shows the detection results by the proposed method. (**a**) car road and a traffic light, (**b**) a bear, (**c**) a kite, (**d**) detection result of manipulated image with car road and a zebra, (**e**) manipulated image with a horse and a bat, (**f**) manipulated image with a cow in the sky.

The experimental results for the natural images (the positive set) and forged images (the negative set) are shown in Figure 11 and Figure 12. The natural images are from the COCO 2014 test database. Because no manipulation was detected, we showed the output value for which the CL-CNN input contained all the object sets P extracted by the detector. For the natural image, the average output value was 0.98 or higher. For instance, a combination of vases, indoor tables and chairs is frequently observed in the COCO dataset, thus all of them are judged to be natural objects, as shown in Figure 11a. Note that, the appearance rate of people in the training dataset was high; thus, an image in which people appear tends to be evaluated positively by CL-CNN. Therefore, the output values are larger in comparison, as shown in Figure 11b,c.

We created images that were manipulated by using an arbitrary combination of object classes (see Figure 12). Figure 12d, which displays a zebra surrounded by cars and trees, constitutes a contextual abnormality. The yellow box shows the normal detection results by R-CNN, and red box represent the detection result by our method. Because this combination of labels is rare (or does not exist), the output value of the CL-CNN is rather low than in the positive cases. The results confirm that the “naturalness of the image” would be enhanced by removing the zebra.

However, our framework has some limitations. For example, the image in Figure 12f is manipulated by placing a cow beside a kite. However, our method loses the information that the cow is in the sky during the object detection step. Therefore, both the cow and the kite are judged to appear unnatural in the image. In Figure 12e, both the horse and baseball bat were inserted into the image as the result of manipulation, but only the baseball bat was selected as an abnormal object. In this case, other forgery detectors such as [21,22,25] could be combined with our method to improve the detection accuracy.

### 5.3. Detection Complement with Other Detectors

In this section, review sample results that show that other forgery detectors could be combined with our method to improve the detection performance. Low-level image feature based techniques [9,21,25,35] can help to complement our method. The low-level image feature such as noise, resampling and JPEG artifacts are severely damaged by various post-processing techniques, so their detection performance will be further improved by our method. Although other forensic tools can detect the objects our method misclassified, our method can also be used for enhancing the other forensic tools.

**Figure 13:**
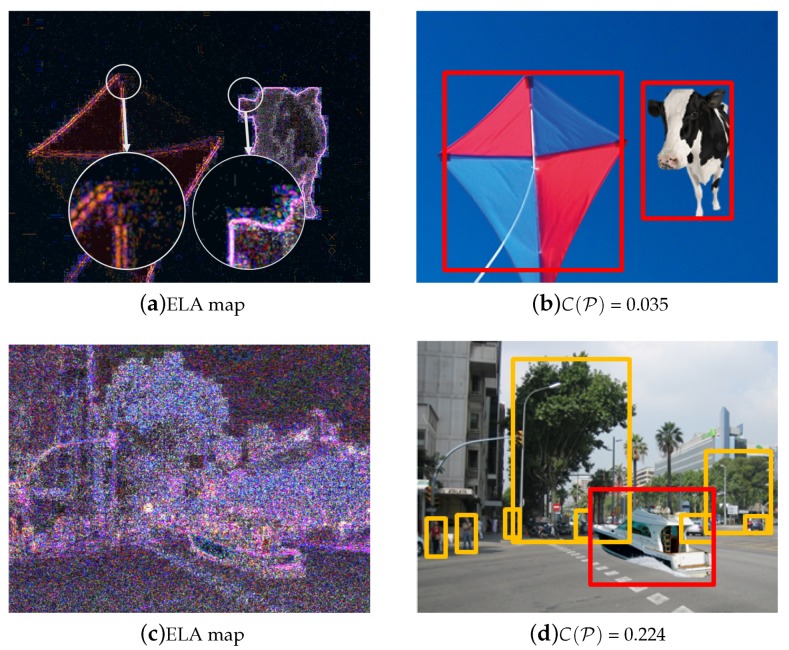
Error level analysis (ELA) highlights differences in the JPEG compression level. (**a**) ELA boost high-contrast edges from the cow that may have been digitally changed, but (**b**) the inserted cow cannot be distinguished by our detector. As an opposite sample, (**c**) ELA is unable to reveal the changed region when the image has been globally post-processed. (**d**) the proposed method confirms that a boat is out-of-context. The yellow box shows the normal detection results by R-CNN, and red box represent the detection result by our method.

For instance, error level analysis (ELA) identifies areas within an image that are at different compression levels [35]. ELA highlights differences in the JPEG compression level because re-saving a JPEG removes high-frequency patterns and results in less differences between high-contrast edges and textures. As shown in Figure 13a,b, the inserted cow cannot be distinguished by our detector, but ELA boosts high-contrast edges from the cow that may have been digitally changed. However, if the modified area of the image is globally processed with strong compression, noise addition or resizing, ELA is unable to reveal the changed region of the image. As we can see in Figure 13d, our method can detect such forgeries even after the strong post-processing, although ELA is unable to reveal the changed region when the image has been globally post-processed.

**Figure 14:**
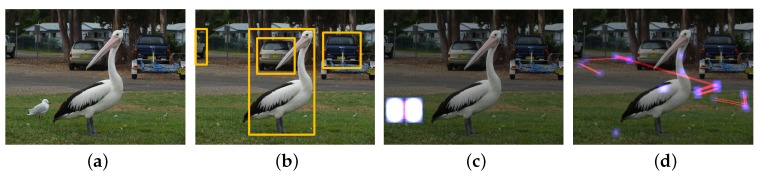
A part of grass image was copied and covered into the part of a seagull to make it disappear from the image. (**a**) Original image; (**b**) object detection result of the manipulated image; (**c**) detection result of the copy–move detector; (**d**) false positives of the detector on post-processed image.

Another family of forgery is a copy–move modification [9,10,11]. In a copy–move forgery, a part of the image itself is copied and pasted into another part of the same image [9]. This is usually performed with the intention to make the targeted object disappear from the image by covering it with a segment copied from another part of the image. (See a seagull on the grass in Figure 14a. Copy–move forgery detector only targets a specific type of image modification. Similar to ELA, a copy–move detector generally fails when the suspicious image has been post-processed, such as resizing, as shown in Figure 14d.

As we have seen so far, each detector has its own specified forgery and counterfeit method. Because the basics of our method differs significantly from other methods, the proposed detector is useful to complement the drawbacks of the existing forensic techniques.

## 6. Discussion and Conclusions

In this study, we proposed a model (CL-CNN) that can provide contextual information prior to directly learning the combination of image labels. The trained model provides a contextual prior based on CNNs. The proposed method first uses a well-known object detector such as R-CNN [30] to detect the class and location of the object and then evaluates the contextual scores based on the combination of objects.

The proposed method can be used for various applications. Because many of the available forensic tools require a large amount of computational power, this method can be used to pre-filter web-scale image candidates. The abnormality detector could be used in a specific application to monitor an image server or a large database. Unlike low-level feature-based forgery detectors in which forgery detection performance is degraded by common image processing techniques, our method can detect forgery by measuring abnormality if only objects are identified. Thus, our method complements other forgery detection techniques, especially those that are low-level feature-based, such as photo response non-uniformity (PRNU) [5], camera response function [24] and JPEG compression trace [25]. Our method is a learning-based technique, so the application can be easily extended and fine-tuned with any other database.

However, the region-based object detector used in this study ignores the background of an image; hence, our method does not allow the context between the object and its background to be directly evaluated. Therefore, we plan to enhance the accuracy of the study by combining deep learning based on scene classification, similar to the approach that was followed in recent studies [36,37]. In addition, we aim to improve the model to obtain more robust and accurate results by paying more attention to the generation of negative sets.
